# Quantum teleportation mediated by surface plasmon polariton

**DOI:** 10.1038/s41598-020-67773-1

**Published:** 2020-07-13

**Authors:** Xin-He Jiang, Peng Chen, Kai-Yi Qian, Zhao-Zhong Chen, Shu-Qi Xu, Yu-Bo Xie, Shi-Ning Zhu, Xiao-Song Ma

**Affiliations:** National Laboratory of Solid-State Microstructures, School of Physics, Collaborative Innovation Center of Advanced Microstructures, Nanjing University, Nanjing, 210093 China

**Keywords:** Nanophotonics and plasmonics, Quantum optics

## Abstract

Surface plasmon polaritons (SPPs) are collective excitations of free electrons propagating along a metal-dielectric interface. Although some basic quantum properties of SPPs, such as the preservation of entanglement, the wave-particle duality of a single plasmon, the quantum interference of two plasmons, and the verification of entanglement generation, have been shown, more advanced quantum information protocols have yet to be demonstrated with SPPs. Here, we experimentally realize quantum state teleportation between single photons and SPPs. To achieve this, we use polarization-entangled photon pairs, coherent photon–plasmon–photon conversion on a metallic subwavelength hole array, complete Bell-state measurements and an active feed-forward technique. The results of both quantum state and quantum process tomography confirm the quantum nature of the SPP mediated teleportation. An average state fidelity of $0.889\pm 0.004$ and a process fidelity of $0.820\pm 0.005$, which are well above the classical limit, are achieved. Our work shows that SPPs may be useful for realizing complex quantum protocols in a photonic-plasmonic hybrid quantum network.

## Introduction

The hybrid light-matter nature of surface plasmon polaritons (SPPs) allows light to be confined below the diffraction limit, opening up the possibility of subwavelength photonic device integration^1^. The quantum properties of SPPs originate from quantized surface plasma waves, and several quantum models have been proposed to describe the electromagnetic field of a plasmon^2,3^. The quantization of SPPs has motivated many researchers to explore the fundamental quantum phenomena associated with them, for example, plasmon-assisted transmission of entangled photons^4,5^, single-plasmon state generation and detection^6,7^, quantum statistics and interference in plasmonic systems^8–13^, quantum logic operations^14^, anti-coalescence of SPPs in the presence of losses^15^ and quantum plasmonic N00N state for quantum sensing^16^. For reviews, see Ref.^17,18^. Recently, some quantum properties of new plasmonic metamaterials have also been explored, such as coherent perfect absorption in plasmonic metamaterials with entangled photons^19^, testing hyper-complex quantum theories with negative refractive index metamaterials^20^ and the active control of plasmonic metamaterials operating in the quantum regime^21^.

These works motivate us to study and utilize the quantum properties of SPPs in more advanced quantum information protocols. Quantum teleportation uses entanglement as a resource to faithfully transfer unknown quantum states between distant nodes. Ever since it was first introduced by Bennett et al.^22^ and experimentally realized using photonic qubits^23,24^, quantum teleportation has become the essential protocol for establishing worldwide quantum networks^25,26^. The teleportation distance has increased significantly over the last two decades^27–31^ and has recently been successfully extended to more than a thousand kilometres from the ground to a satellite^32^. To build a quantum network with more functionalities, various physical systems are required with individual advantages in terms of transferring and processing the quantum state.

## Results

### The conceptual scheme of SPP mediated quantum teleportation

**Figure 1:**
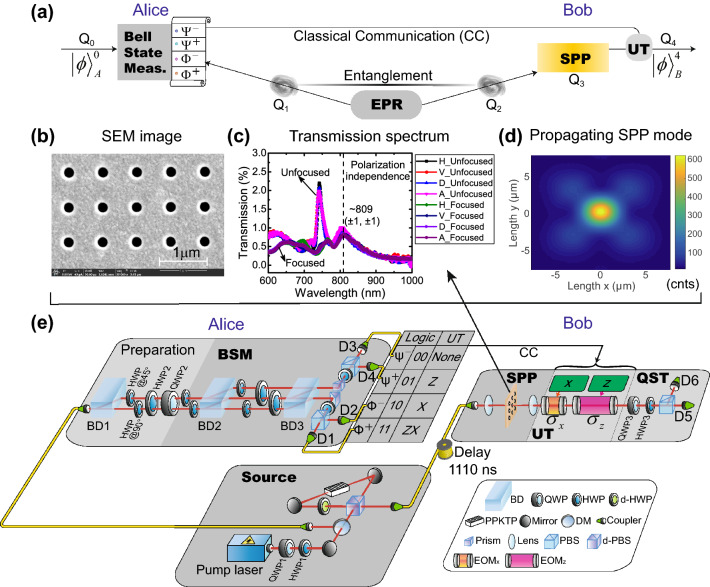
Experimental layout of the surface plasmon polariton (SPP) mediated quantum teleportation. (**a**) The conceptual framework of our experiment. At Alice’s site, the input states are prepared using qubit 0 (Q0). An Einstein-Podolsky-Rosen (EPR) source generates two entangled qubits, Q1 and Q2. Q1 is sent to Alice for a Bell-state measurement (BSM)^24^. Q2 is sent to Bob to excite the SPP qubit, Q3. Through the photon–plasmon–photon conversion, the quantum states of the SPPs are transformed back to a photonic qubit, Q4. The outcomes of the BSM are sent to Bob using the classical communication (CC). Bob then applies a unitary transformation (UT) to Q4. As a result, the output state $|\phi \rangle ^4_B$ is identical to $|\phi \rangle ^0_A$; hence, teleportation is accomplished. (**b**) The SEM image of the subwavelength hole arrays with 200 nm diameter and 700 nm period. (**c**) Transmission spectrum of the hole arrays. The resonance at approximately 809 nm (dashed line) is the ($\pm 1$,$\pm 1$) mode, corresponding to the SPPs propagating along the diagonal direction. (**d**) The far-field image shows the SPP propagation mode. The units ‘counts’ (cnts) is labelled below the colorbar. (**e**) Sketch of the experimental setup. The polarization-entangled source uses a type-II down-conversion Sagnac interferometer, where a $\chi ^{(2)}$ nonlinear crystal (periodically poled $\hbox {KTiOPO}_4$, PPKTP) is coherently pumped by 405 nm laser light from clockwise and counter-clockwise directions. The central wavelength of the entangled signal (A) and idler (B) photons is approximately 810 nm. Photon A is sent to Alice. The polarization degree of freedom (DOF) (Q0) of photon A is used for preparing the six input states. The four Bell states are constructed using the path (Q1) and polarization (Q0) DOF of photon A. Photon B is sent to Bob. The polarization of photon B (Q2) is used to excite the SPPs. After undergoing a photon–plasmon–photon conversion, the quantum state of the SPPs (Q3) is transferred back to the photon (Q4). The results of the BSM (00, 01, 10, 11) are sent to Bob by CC and subsequently used to trigger the electro-optic modulators (EOMs, $\sigma _x$, $\sigma _z$) to apply the corresponding UTs ($I, \sigma _z, \sigma _x, i\sigma _y$). The quantum state is finally analysed through quantum state tomography (QST). HWP: half-wave plate; QWP: quarter-wave plate; BD: beam displacer; DM: dichromatic mirror; d-PBS: dual-wavelength polarizing beam splitter.

We experimentally realize the quantum state teleportation of a single photon to a single SPP, which is a single qubit consisting of collective electronic excitations typically involving ${\sim }10^{6}$ electrons^17^. Our scheme is based on three qubits, which is first proposed by Popescu^33^ and realized in experiment by Boshi et al.^24^. The conceptual framework of our experiment with the three-qubit scheme is shown in Fig. 1a. The entanglement between qubits 1 (Q1) and 2 (Q2), serving as the quantum channel, is generated from the entangled photon-pair source and distributed to Alice and Bob. An input state of qubit 0 (Q0) is sent to Alice. Alice performs a Bell-state measurement (BSM)^24^, projecting Q0 and Q1 randomly into one of the four Bell states, each with a probability of 25%. Then, the outcomes of the BSM are sent to Bob through a classical communication (CC) channel. Q2 is sent to a subwavelength hole array sample patterned on a gold film at Bob’s site to facilitate the photon–SPP–photon conversion^34^. There, the quantum state of Q2 is transferred to qubit 3 (Q3), carried by a single SPP. This SPP propagates along the surface of the sample and subsequently couples to an optical photon (Q4), which radiates towards detectors in the far field. According to the outcomes of the BSM, the corresponding unitary transformations (UTs) are applied to Q4. Finally, we perform quantum state tomography (QST)^35,36^ on Q4 and verify whether the quantum state teleportation from a single photon to a single SPP is successful by evaluating the quantum state fidelities of Q4 to Q0 and the quantum process fidelity of the whole procedure.

### Subwavelength hole array and its characterization

Figure 1b shows a scanning electron microscopy (SEM) image of the subwavelength hole array used in our experiment. The gold film is perforated over a square area of $189\times 189\, \upmu \hbox {m}^2$ with periodic hole arrays by using a focused ion beam. The hole diameter and the period are 200 nm and 700 nm, respectively. The thickness of our metal film is 150-nm. Although the hole array reduces the direct photon transmission, it allows resonant excitation of the SPP^34^.

The transmission spectrum of our sample is shown in Fig. 1c and has a peak centred at approximately 809 nm with a full width at half maximum (FWHM) of ${\sim }70\hbox { nm}$. The peak transmittance of the sample at 809 nm is approximately 0.8%. The extraordinary optical transmission (EOT) observed in the subwavelength hole arrays is a typical resonant tunneling phenomenon which results from the constructive interference when the photons go through the holes^34,37^. Compared with other works^4,34^, the total transmittance of our sample is slightly lower. The reason is that the transmission spectrum is very sensitive to the geometrical parameters of the system^37,38^. The imperfections during the fabrication can lead to the hole shape, period of the lattice as well as thickness and smoothness of the gold film departure from the nominal settings, thus resulting in the low transmission^39^. Even setting the same parameters, the transmission of samples fabricated at different times has some obvious differences and is lower than 3% due to the fabrication imperfections^4^. However, we only utilize the frequency information, i.e. peak position, instead of the transmittance in our teleportation experiment. Although our overall transmission is smaller than 2.5%, it is still larger than the value predicted by the standard aperture theory^34^, which indicates that the EOT does happen in our sample. The transmission curves for different light polarizations are similar, indicating that our sample is nearly polarization-independent. The polarization insensitivity is due to the symmetry of the square lattice, as have been demonstrated in previous works^38,40,41^. A numerical calculation based on the geometry of the array and the wavevector matching shows that this peak is associated with the ($\pm 1,\pm 1$) SPP modes at the glass-metal interface^42^. These modes can excite the SPPs propagating along the four diagonal directions. We experimentally measure the SPP propagation with a laser and a charge-coupled device (CCD), as shown in Fig. 1d. By fitting to the SPP propagation along the diagonal direction, we estimate the 1/*e* decay length of the plasmonic mode to be ${\sim }4.48\pm 0.50\, \upmu \hbox {m}$. See the Supplementary Information for more details on the numerical simulation, design of the hole array and characterizations of this device.

### Realizing quantum teleportation between photon and SPP

Figure 1e presents a layout of our experimental setup. The entangled photon pairs are generated from spontaneous parametric down conversion, which is realized by embedding a periodically poled $\hbox {KTiOPO}_4$ (PPKTP) crystal in a Sagnac interferometer^43,44^. The quantum state of photons A and B is similar to the singlet state:

1 $$\begin{aligned} |\Psi ^{-}\rangle _{AB} = \frac{1}{\sqrt{2}}(|H\rangle _{A}|V\rangle _{B}-|V\rangle _{A}|H\rangle _{B}), \end{aligned}$$

which has a fidelity of approximately $98\%$. $|H\rangle _{A}$ ($|V\rangle _{A}$) denotes the horizontal (vertical) polarization state of photon A. The same notation is used for photon B. We obtain coincidence counts at a rate of approximately 100 kHz with a pump power of 20 mW.

We employ the two-photon three-qubit scheme to realize the SPP mediated quantum teleportation^24,29^. The two-photon three-qubit scheme has the advantages that it avoids the very low detection rates caused by the simultaneous detection of three photons and allows a 100% Bell state measurement^24,29,33^. We note that two-photon scheme of teleportation has limitation as one can’t use this scheme to teleport the quantum state of an independent photon which comes from outside. In our experiment, photons A and B are sent to Alice and Bob through single-mode fibre (SMF), respectively. We use photon A’s polarization as Q0 and its path state as Q1. Photon B’s polarization acts as Q2. First, we swap the entanglement between Q0 and Q2 (see Eq. (1)) to Q1 and Q2. We achieve this by sending photon A through a beam displacer (BD1 in Fig. 1e), which makes the horizontal polarized component undergo a lateral displacement into the left path mode (denoted as $|l\rangle$) and transmits the vertically polarized component directly (denoted as $|r\rangle$). The two-photon (A and B) three-qubit (Q0, Q1 and Q2) state can be written as

2 $$\begin{aligned} |\Psi ^{-}\rangle ^{012}_{AB} = \frac{1}{\sqrt{2}}(|H\rangle ^{0}_{A}|l\rangle ^{1}_{A}|V\rangle ^{2}_{B} -|V\rangle ^{0}_{A}|r\rangle ^{1}_{A}|H\rangle ^{2}_{B}). \end{aligned}$$

Note that the superscripts are labelled for the qubit and the subscripts are labelled for the photon. Then, a $45^\circ$-oriented HWP (HWP@45$^\circ$ in Fig. 1e) rotates the horizontal component ($|H\rangle _A$) to the vertical polarization ($|V\rangle _A$) in the left path, $|l\rangle$. Along the right path, $|r\rangle$, a $90^\circ$-oriented HWP (HWP@90$^\circ$ in Fig. 1e) is used for phase compensation. After these two HWPs, the polarization state of photon A (qubit 0) is in $|V\rangle$ and is factorized out. The full state is as follows:

3 $$\begin{aligned} |\Psi ^{-}\rangle ^{012}_{AB} = \frac{1}{\sqrt{2}}|V\rangle ^{0}_{A}\otimes (|l\rangle ^{1}_{A} |V\rangle ^{2}_{B}-|r\rangle ^{1}_{A}|H\rangle ^{2}_{B}). \end{aligned}$$

Consequently, the initial entanglement between the polarization states of photons A and B is swapped into the path state of photon A (qubit 1) and the polarization state of photon B (qubit 2)^45,46^.

The combination of HWP2 and QWP2 are then used to create the polarization state to be teleported (see Sect. S5 of Supplementary Information), i.e. $|\phi \rangle ^{0}_{A}=\alpha |H\rangle ^{0}_{A}+\beta |V\rangle ^{0}_{A}$, where $\alpha$ and $\beta$ are two complex numbers satisfying $|\alpha |^2+|\beta |^2=1$. This process can be expressed as follows:

4 $$\begin{aligned} |\Psi ^{-}\rangle ^{012}_{AB}= & {} \left( \alpha |H\rangle ^{0}_{A}+\beta |V\rangle ^{0}_{A}\right) \otimes \frac{1}{\sqrt{2}}\left( |l\rangle ^{1}_{A}|V\rangle ^{2}_{B}- |r\rangle ^{1}_{A}|H\rangle ^{2}_{B}\right) \nonumber \\= & {} \frac{1}{2}\left( i\sigma _y|\phi \rangle ^{2}_{B}|\Phi ^{+}\rangle ^{01}_{A} +\sigma _x|\phi \rangle ^{2}_{B}|\Phi ^{-}\rangle ^{01}_{A}-\sigma _z |\phi \rangle ^{2}_{B}|\Psi ^{+}\rangle ^{01}_{A}+I|\phi \rangle ^{2}_{B} |\Psi ^{-}\rangle ^{01}_{A}\right) \end{aligned}$$

Here the polarization (Q0) and path states (Q1) of photon A are used to construct the four Bell states: $|\Psi ^{\pm }\rangle ^{01}_A=\frac{1}{\sqrt{2}}(|V\rangle _{0}|l\rangle _{1} \pm |H\rangle _{0}|r\rangle _{1})$ and $|\Phi ^{\pm }\rangle ^{01}_A=\frac{1}{\sqrt{2}}(|H\rangle _{0}|l\rangle _{1} \pm |V\rangle _{0}|r\rangle _{1})$. Alice realizes a complete BSM using the polarization (Q0) and path (Q1) DOF of photon A with BD2 and BD3 (see Sect. S5 of Supplementary Information for details). The outcomes of the BSM are sent from Alice to Bob via coaxial cables.

Photon B (Q2) is delayed by a 222-m-long (corresponding to a temporal delay of ${\sim }1110$ ns) SMF and then sent to Bob. At Bob’s site, Q2 is focused on the subwavelength hole arrays and converted to a single surface plasmon (Q3). As a result, we coherently transmit the quantum state of Q2 to Q3, which is carried by the single-mode collective electronic excitations of the SPP. Then, the SPP propagates along the surface of the sample and subsequently couples out to an optical photon (Q4), radiating into the far field. After the BSM is performed by Alice, the quantum state of Q4 is projected into a pure state and equals the input state $|\phi \rangle ^{0}_{A}$ up to a local UT according to the BSM result (see Eq. (4)). The local UTs are realized with two EOMs, which perform the required $\sigma _x$ and $\sigma _z$ operations. Collectively, the EOMs perform the $i\sigma _y$ operation. After these local UTs, the output state of Q4 is: $|\phi \rangle ^{4}_{B}=\alpha |H\rangle ^{4}_{B}+\beta |V\rangle ^{4}_{B}$. Finally, we collect the photons into an SMF and perform QST on Q4.

### The results of quantum state and process tomography

**Figure 2:**
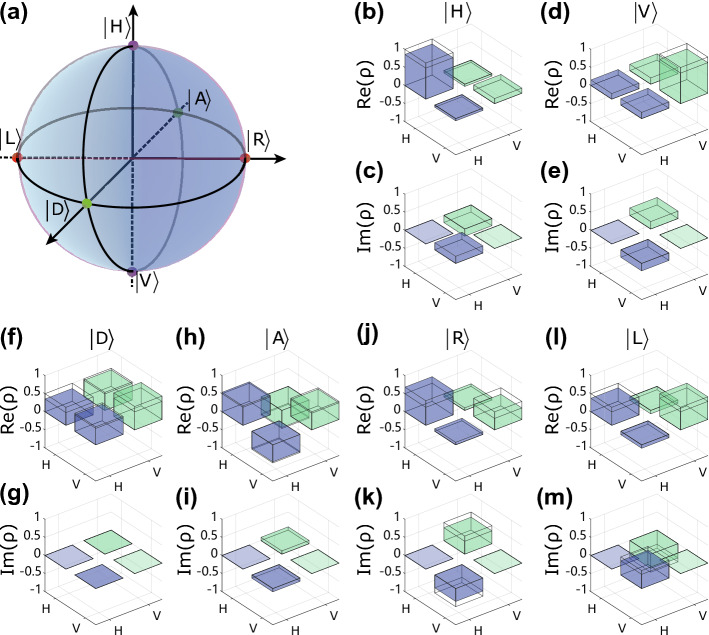
Reconstructed density matrices of the six teleported states. (**a**) The initial prepared states are $|H\rangle$, $|V\rangle$, $|D\rangle$, $|A\rangle$, $|R\rangle$, and $|L\rangle$ and are indicated by coloured dots on the Bloch sphere. (**b**,**d**,**f**,**h**,**j**,**l**) Real parts of the reconstructed density matrices for the six states. (**c**,**e**,**g**,**i**,**k**,**m**) Imaginary parts of the reconstructed density matrices for the six states. The ideal density matrix is shown as the wire grid. The representative data here are for experiments with a $|\Phi ^{+}\rangle$ Bell-state measurement outcome with SPP. The reconstructed density matrices of the six states for all four Bell-state measurement outcomes are provided in the Supplementary Information.

We prepare six input states of qubit 0: $|H\rangle$, $|V\rangle$, $|D\rangle$, $|A\rangle$, $|R\rangle$, and $|L\rangle$ (see Fig. 2a). Note that $|D\rangle =(|H\rangle +|V\rangle )/\sqrt{2}$/$|A\rangle =(|H\rangle -|V\rangle )/\sqrt{2}$, and $|R\rangle =(|H\rangle -i|V\rangle )/\sqrt{2}$/$|L\rangle =(|H\rangle +i|V\rangle )/\sqrt{2}$ stand for the diagonal/anti-diagonal linearly and right/left circularly polarized states of single photons, respectively.

**Figure 3:**
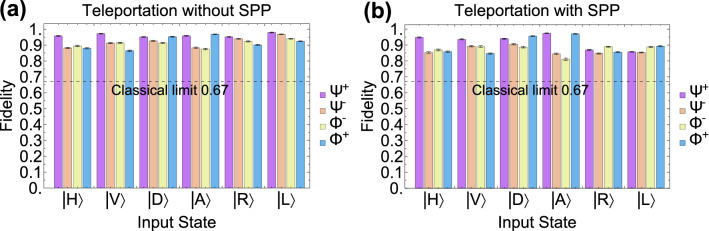
Quantum state fidelities of quantum teleportation for the six different input states: $|H\rangle$, $|V\rangle$, $|D\rangle$, $|A\rangle$, $|R\rangle$ and $|L\rangle$ with four Bell-state measurement results: $|\Psi ^{-}\rangle$, $|\Psi ^{+}\rangle$, $|\Phi ^{-}\rangle$ and $|\Phi ^{+}\rangle$. The different BSM outcomes are denoted with different colours. (**a**) The fidelities measured without the SPP involved. We perform this measurement by moving the subwavelength hole array out from the setup. (**b**) The fidelities measured with the SPP involved. All the fidelities exceed the classical limit of 2/3 (dashed line). The error bars are calculated using a Monte Carlo routine assuming Poissonian statistics.

To characterize the quantum teleportation mediated by the SPP, we perform single-qubit QST measurements on the teleported quantum states. In Fig. 2b–m, we show the real and imaginary parts of the reconstructed density matrices for different input states. With the reconstructed density matrices, we calculate the state fidelity $F={}_{ideal}{\left\langle \phi \right| }\rho |\phi \rangle _{ideal}$, where $\rho$ is the reconstructed density matrix and $|\phi \rangle _{ideal}$ is the ideal quantum state. The results of the quantum state fidelity after quantum teleportation are shown in Fig. 3. For a comparison, we present the state fidelities both without and with photon–SPP–photon conversion. We can see from Fig. 3 that all the fidelities are well above the limit of 2/3 that can be achieved using a classical strategy without employing entanglement^47^. By averaging the single photon fidelities over all input states, we obtain an average fidelity of $92.67\pm 0.32\%$ (without SPP) and $88.91\pm 0.38\%$ (with SPP) for the retrieved initial states, including active feed-forward operations, which exceed the classical limit of 2/3 by more than 81-$\sigma$ and 58-$\sigma$ standard deviations^47^. We note that the difference in the state fidelities between the cases without the SPP and with the SPP is mainly caused by: The excited SPP distorts the beam pattern and then leads to a lower contrast of the phase flip of the two EOMs. Quantitative analysis of the reduction in the achievable fidelity can be found in the Supplementary Information (Sect. S7).

**Figure 4:**
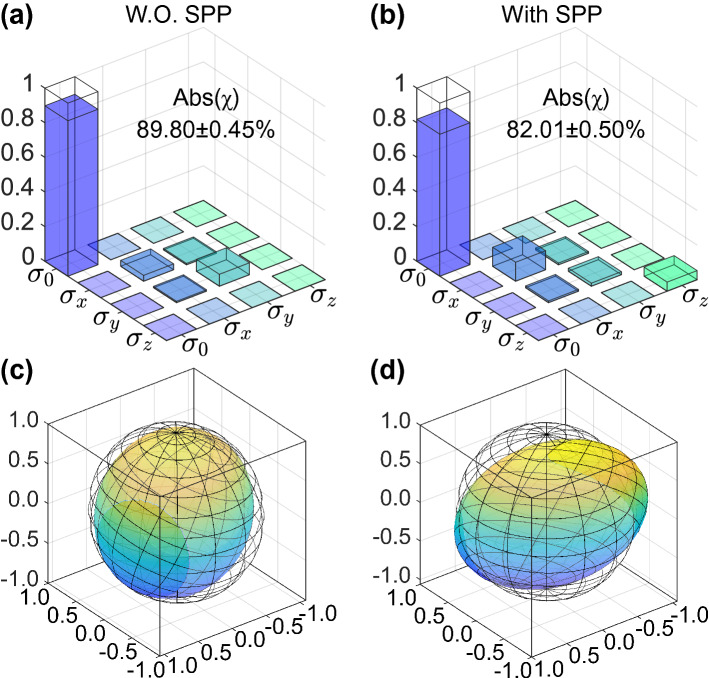
Results of quantum process tomography for the teleportation procedure. (**a**) The real part of the reconstructed process matrix $\chi$ without the SPP (W.O. SPP). The ideal process matrix has only one nonzero component $(\chi _{ideal})_{00}$=1, and we obtain a process fidelity of $F_{proc}=\text {Tr}(\chi _{ideal}\chi )=(89.80\pm 0.45)\%$. (**b**) The real part of the reconstructed process matrix $\chi$ with the SPP (With SPP). The process fidelity is $F_{proc}=\text {Tr}(\chi _{ideal}\chi )=(82.01\pm 0.50)\%$. (**c**,**d**) Bloch sphere representations of the process without (W.O.) (**c**) and with (**d**) the SPP involved. The plot shows how the input states lying on the surface of the initial Bloch sphere (meshed surface) are transformed by our teleportation protocol, with the output states lying on the solid surface.

Since quantum teleportation is a quantum process, it is natural to quantitatively describe the whole process with quantum process tomography^48^. The reconstructed density matrices of the teleported quantum states allow us to fully characterize the teleportation procedure by quantum process tomography. We choose four input states ($\rho _{in} = |H\rangle \langle H|, |V\rangle \langle V|, |D\rangle \langle D|, |L\rangle \langle L|$) and their corresponding output states $\rho _{out}$ to benchmark the process of quantum teleportation. The effect of teleportation on $\rho _{in}$ is determined by the process matrix $\chi$, which is defined by $\rho _{out} = \sum _{l,k = 0}^3 \chi _{lk} \sigma _l \rho _{in} \sigma _k$, where $\sigma _{i}$ are the Pauli matrices with $\sigma _{0}$ being the identity operator. A perfect process matrix of quantum teleportation has only one nonzero component, $\chi _{00}=1$, indicating that the input state is faithfully teleported without a reduction in the state fidelity. The real parts of the process matrix $\chi$ for the two situations (without and with the SPP) are shown in Fig. 4a (without SPP) and Fig. 4b (with SPP), respectively. The quantum process fidelities, i.e. $\mathscr {F}_{proc}=\text {Tr}(\chi _{ideal}\chi )$, for our experiment without and with the SPP are 0.898±0.005 and 0.820±0.005, respectively. These fidelities correspond to 80-$\sigma$ and 64-$\sigma$ violations over the classical bound of 0.5^31,49^. A single-qubit quantum process, including quantum teleportation, can be represented graphically by a deformation of the Bloch sphere subjected to the quantum process^48^. As shown in Figs. 4c (without SPP) and d (with SPP), the ideal input states of Q0 are denoted as the states lying on the meshed surface of the Bloch sphere. After the photon-to-SPP quantum teleportation, the initial Bloch spheres are deformed into anisotropic ellipsoids as shown in the solid blue-yellow colour, corresponding to the final output states.

## Discussion

Note that the transmission losses reduce the coincidence count rate in our experiment. Therefore, we have to increase the integration time to obtain enough coincidence counts (see Table S7 in the Supplementary Information) for obtaining statistical significance. However, the advantages of plasmonic systems are that they are very suitable for making miniaturized quantum devices. Their sizes can be reduced such that the quantum logic operations can be finished below the propagation distances before plasmons are lost^14^. Recently, the new techniques of material growth and structure design have helped in greatly minimize or mitigate the influence of losses on the plasmonic devices^50–52^. In some cases, the losses can provide new insights into quantum physics, such as the lossy beam splitter exhibiting fermionic anti-coalescence behavior using surface plasmons^15,53^.

In our present experiment, the average teleportation fidelity for the retrieved initial states is smaller for the case with SPP than that without SPP. As have been explained in Sect. S7 of the Supplementary Information, the reduction of fidelity is caused by the low contrast of feed-forward operations. With EOMs of higher extinction ratio, it is possible to improve the fidelity. In addition, the fabrication techniques can also be optimized to improve the quality of the sample and alleviate the deterioration of the light beam. We would like to elaborate on the motivation of our work from two different perspectives: 1, From a fundamental perspective: although quantum teleportation has been demonstrated with many different physical systems, to the best of our knowledge, it has never been implemented with the plasmonic system, a system consisting of $10^6$ electrons. It would be interesting to see if the quantum teleportation could work between two systems with such dramatic particle number difference, namely one photon vs $10^6$ electrons. 2, From the application perspective, plasmonic devices allow us to implement quantum operation with orders-of-magnitude smaller dimension^14^ comparing to $\hbox {SiO}_2$ or Si integrated devices^54^. However, to realize more complicated quantum information protocols, such as quantum teleportation of quantum gates^55^ or quantum teleportation based quantum computation^56^ with plasmonic systems, we have to firstly experimentally verify the feasibility of quantum teleportation via plasmons. We view our work as the decisive step towards that goal, as the average state fidelity of the teleportation mediated by SPP we obtained exceeds the classical limit of 2/3 by more than 58-$\sigma$ standard deviations, which shows the plasmonic system is robust against the reduction of fidelity.

In summary, we demonstrate faithful teleportation of quantum states from one qubit of a single photon to another qubit of an SPP. The photon-to-SPP quantum teleportation is completely characterized by quantum state and process tomography. The fidelities of the six teleported states all exceed the classical limit with tens of standard deviations. The process fidelities also exceed the classical limit with tens of standard deviations. These results conclusively confirm the quantum nature of teleportation from arbitrary unknown quantum states of a single photon to a single SPP. Our work is a further step towards exploring the fascinating quantum behaviours of SPPs. The comprehensive utilization of the quantum properties of SPPs in more advanced protocols will promote the rapid development of future quantum information processing with quantum plasmonic devices.

## Supplementary information

Supplementary information

## Data Availability

The data used in current study are available from the corresponding author upon reasonable request.
