# Supplementary material for: Quantum teleportation mediated by surface plasmon polariton
Source: Sci Rep. 2020 Jul 13;10:11503. doi: 10.1038/s41598-020-67773-1 (PMC7359310; doi:10.1038/s41598-020-67773-1)
Supplement: Supplementary file 1 — Supplementary information [file 41598_2020_67773_MOESM1_ESM.pdf]

SUPPLEMENTARY INFORMATION: Quantum teleportation mediated by surface plasmon polariton

Xin-He Jiang[1,†], Peng Chen[1,†], Kai-Yi Qian[1,†], Zhao-Zhong Chen[1], Shu-Qi Xu[1], Yu-Bo Xie[1], Shi-Ning Zhu[1], and Xiao-Song Ma[1,*]

[1]National Laboratory of Solid-state Microstructures, School of Physics, Collaborative Innovation Center of Advanced Microstructures, Nanjing University, Nanjing 210093, China
[*]Xiaosong.Ma@nju.edu.cn
[†]These authors contributed equally to this work

Contents

S1 FDTD simulation and design of the sample 2
S2 Experimental characterization of the sample 4
S3 Source of entangled photons 4
S4 Certification of quantum properties of SPP 5
S5 Phase control of Bell-state analyser 5
S6 Feed-forward unitary transformations 7
S7 Reconstructed density matrix and teleportation fidelity 11
References 14

S1 FDTD simulation and design of the sample

To design the hole array with proper peak position and transmittance, we thoroughly perform finite-difference time-domain (FDTD) simulations with the commercial software Lumerical (FDTD solution, Lumerical Inc.). The mesh size of the primitive cell is 10 nm to ensure the accuracy of electromagnetic field calculations within the gold layer. The simulation model is shown in Fig. S1. The FDTD region is set to have the period p in both x and y direction, and 10 μm in z direction. The thickness of the gold layer is set to be t. The hole is perforated as a cylinder of diameter d. To provide a comprehensive guide to the fabrication, we simulate several periods (680, 690, 700, 710, 720 nm, etc.), thicknesses (130, 140, 150, 160, 170 nm, etc.) and diameters (180, 190, 200, 210, 220 nm, etc.). The boundary conditions in x and y directions are 'periodical' and the z direction uses the 'Perfectly Matched Layer (PML)' boundary condition. The broadband light source is illuminated from the air-metal interface and the monitor placed at 5 μm away from the metal-glass interface is used to measure the transmitted light.

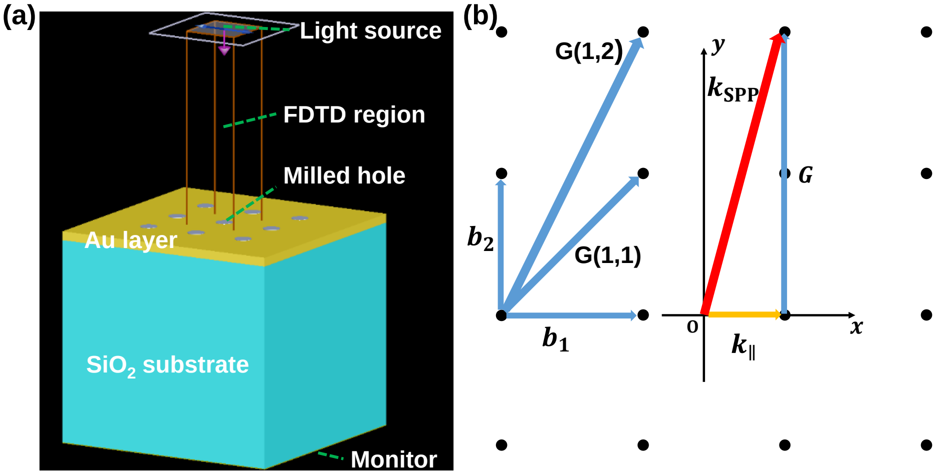

Figure S1. Structure of our simulation model and reciprocal lattice. (**a**) A mesh region of p nm in x and y direction, and 10 μm in z direction is used in the FDTD simulation. (**b**) Reciprocal lattice of hole array. The blue arrows indicate the reciprocal vectors. $\boldsymbol{k}_{\parallel}$ is the in-plane wave vector of the incident light. Excitation occurs when the wave vector of SPP $\boldsymbol{k}_{\text{SPP}}$ matches $\boldsymbol{k}_{\parallel}$ plus one reciprocal vector $\boldsymbol{G}$.

The dispersion relation for the resonant excitation of surface plasmons in a 2D lattice is

$$\begin{aligned} \boldsymbol{k}_{\text{SPP}} &= \boldsymbol{k}_{\parallel} + \boldsymbol{G} \\ \boldsymbol{G}(m_1, m_2) &= m_1 \boldsymbol{b}_1 + m_2 \boldsymbol{b}_2 \end{aligned} \tag{S1}$$

where $\boldsymbol{k}_{\parallel}$ is the in-plane wave vector, $\boldsymbol{k}_{\text{SPP}}$ is the excited surface plasmon wave vector, $\boldsymbol{b}_1$ and $\boldsymbol{b}_2$ are the primitive vectors of reciprocal lattice (see Fig. S1(b)), $|\boldsymbol{b}_1| = |\boldsymbol{b}_2| = 2\pi/p$ with p as the period of the hole array. This relation is guaranteed by the conservation of momentum. It is convenient to denote the wave vector of excited SPP $\boldsymbol{k}_{\text{SPP}}$ as the (m_1, m_2) mode. Generally, we can distinguish these modes according to the resonant wavelength of the excited SPP[1]:

$$\lambda(i,j) = \frac{p}{\sqrt{m_1^2 + m_2^2}} \sqrt{\frac{\varepsilon_{\text{S,A}} \varepsilon_{\text{M}}}{\varepsilon_{\text{S,A}} + \varepsilon_{\text{M}}}} \tag{S2}$$

where m_1 and m_2 are mode indices, ε_{M} is the dielectric constant of the metal, and $\varepsilon_{\text{S,A}}$ is the dielectric constant of substrate/air ($\varepsilon_{\text{S}}/\varepsilon_{\text{A}}$) in contact with the metal. For the sample used in our experiment, we can put the dielectric constants of gold (Au) and SiO_2/air into Eq. (S2) and obtain the mode corresponding to different resonant wavelengths. The calculated results indicate that the wavelength at approximately 810 nm is associated with the $(\pm1, \pm1)$ mode that propagates along the four diagonal directions at the metal-substrate interface, as shown in Fig. 1(d) of main text. We adopt the optical dielectric constant of gold (real part ε_1~-25.8 and imaginary part ε_2~1.0-2.0) and dielectric constant of SiO_2 substrate (2.16-2.31) from the community[1–3]. The resonant wavelength is taken to be 810 nm. The period p is taken as 700 nm. Substituting these parameters into Eq. (S2), we calculate that the value of m_1^2+m_2^2 is approximately 1.90. Because m_1 and m_2 are integer mode index determining the matching reciprocal vector $\boldsymbol{G}$, the only possible mode labelling this resonant peak is (m_1=$\pm$1, m_2=$\pm$1). It should be pointed out that the transmission peak is not contributed by a single mode, the coherent admixture of different modes leads to the

broadening and shift of the peak[4]. For normal incident light and perfect square lattice, the (1, 1) mode, (1, −1) mode, (−1, 1) mode and (−1, −1) mode are degenerate.

Figure S2 gives our simulation results of transmission spectrum using different periods p, diameters d and thicknesses t. From Eq. (S2) we can see that the resonant wavelength increases when increasing the period of the hole array. This is confirmed by our FDTD simulations. The transmission peaks have a redshift as the period increases. It turns out to be an effective method to adjust the peak position by changing the period of the hole array. For the parameters of diameter and thickness, large thickness and small diameter will result in low transmittance and small full width at half maxima (FWHM). When the ratio t/d is fixed, the peak position will not move too much. All the above analytical and numerical calculations give us a comprehensive guide for the design of the hole array. To reduce the photon losses in the single-photon experiment, we require large d, small p and t to increase the light transmittance. To obtain a peak around 810 nm, the period needs to be set at about 700 nm. To observe a clear $(\pm1,\pm1)$ excitation mode, we require small d and large t to reduce the FWHM to prevent the mixture from other modes.

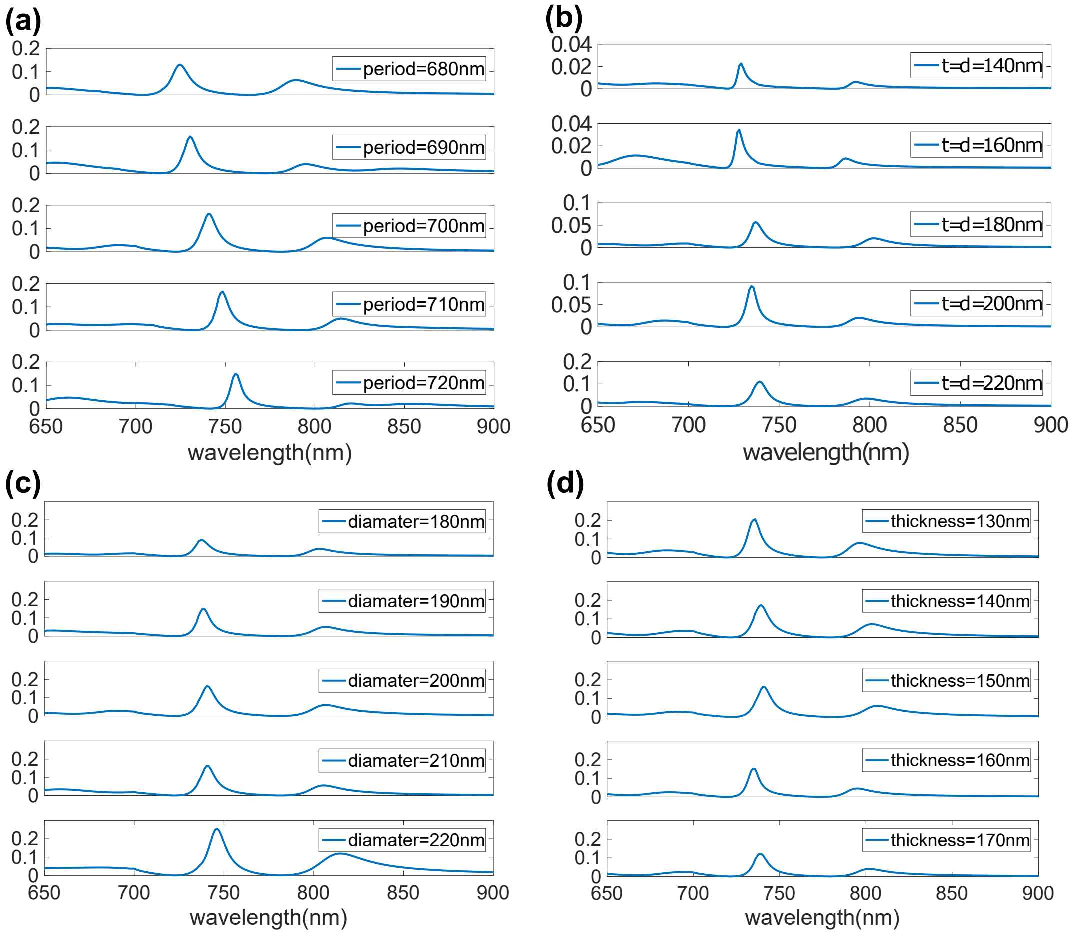

Figure S2. Simulation results for the influence of period, thickness t and diameter d on the transmission. (**a**), The influence of period on the peak position. We choose several periods around 700 nm. It shows an obvious red-shift when increasing the period. (**b**), (**c**), (**d**), The influence of thickness t and diameter d on the transmission spectra. In (**b**), we fix the ratio of t/d. In (**c**) and (**d**), we change the diameter and thickness. From the figure we can see that the peak position does not change so much as the ratio of t/d remains a constant. The thickness and diameter have minor influences on the peak position, but obviously affect the transmittance.

After theoretical calculations and simulations, we fabricate the sample based on the previous optimized parameters. We use quartz (SiO_2) as the substrate. Because of the poor adhesiveness of gold layer and SiO_2 substrate, a 3-nm-thick titanium bonding layer is deposited on the quartz. A 150-nm-thick gold layer is then deposited on the bonding layer using electron-beam evaporation. By means of focused ion beam (FIB), the hole array with a period of 700 nm and hole diameter of 200 nm is milled on the gold layer. The SEM image of our fabricated sample is shown in Fig. 1(b) of main text. Due to the finite image resolution and limited field of view (FOV), we have to move the sample stage to obtain a larger area of hole arrays. Nine small

FOVs are put together closely to make a 3×3 big array. Each FOV includes 90 periods and is therefore 63×63 μm^2 in area. Consequently, the whole hole array has an area of approximately 189×189 μm^2. We also fabricate other hole arrays with different periods and diameters in our experiment. The sample with 150-nm thickness, 700-nm period and 200-nm diameter has a small FWHM and clear $(\pm 1, \pm 1)$ excitation mode. Moreover, it's transmission spectrum is polarization independent which guarantees that polarization-entangled states can be preserved in our single-photon experiment.

S2 Experimental characterization of the sample

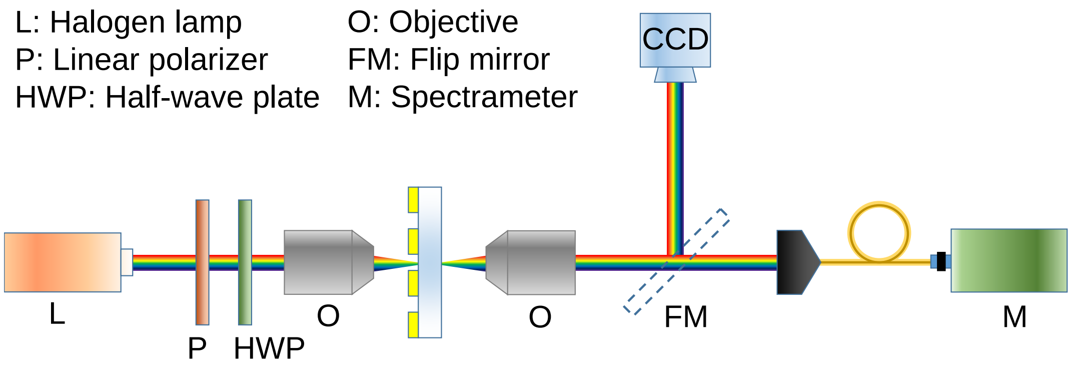

Figure S3. Experimental setup for transmission measurement and the measured and simulated results. The setup for transmission measurement. A white light source is focused on the sample and collected into the spectrometer. We also reflect the light into a charge-coupled device (CCD) for imaging and measuring the SPP excitation mode.

The transmission spectra are measured to characterize the transmission properties of our sample. The experimental setup for the transmission measurement is shown in Fig. S3. We use a polarizer and a half-wave plate (HWP) after a halogen tungsten light source to get a linear polarized light. The sample is focused by a 40× objective. The transmitted light is collected by another 40× objective and collimated into the multimode fibre connected to a spectrometer. For comparison, we also measure the unfocused transmission spectra using a 20× magnification objective (the results are shown in Fig. 1(c) of main text). It is worth mentioning that the ellipticity of holes remarkably influences the transmission spectra of different polarizations. We adjust the focus of FIB and wait for a few minutes to release the stress to improve the circularity of the holes during the fabrication. Figure S4 gives a comparison of the measurement and simulation results for three samples, i.e. t=150, p=700, d=250 (150700250), t=150, p=700, d=200 (150700200) and t=150, p=750, d=200 (150750200). From the figure we can see that the measured peak positions of the transmission spectra are consistent with those of simulations. The peak positions have a redshift when the period varies from 700 to 750 nm. When increasing the diameter of the holes from 200 to 250 nm, the transmittance has an obvious rise. We change the polarization of the incident light and find that the transmission spectra remain the same for the simulation results. For the experiment, the transmission spectra of different polarizations (V and H) have some differences due to the fabrication imperfection of the sample. The 150700200 hole array has an almost identical transmission spectrum for different polarizations and the peak position is around 810 nm, thus it is chosen for the teleportation experiment.

We also measure the SPP excitation mode at 25 different positions of the hole array and get an average figure with a charge-coupled device (CCD) (see Fig. 1(d) in the main text). As shown in Fig. 1(d) of the main text, the four lobes observed along the diagonal/anti-diagonal directions are in accord with the metal-substrate $(\pm 1, \pm 1)$ mode. From the excitation mode, we obtain the SPP propagation distance to be approximately 4.48±0.50 μm ($1/e$ decay length along the diagonal direction, the error is obtained from the standard deviations of the 25 propagation distances). In addition, we also measure the coupling area in the SPP sample using a backward propagation light from the coupler. It turns out that an area with radius of 4.41±0.78 μm can be coupled into the single mode fibre (SMF). This demonstrates that the SPP genuinely participates in the teleportation process.

S3 Source of entangled photons

We use a Sagnac interferometer[5,6] to generate the entangled photons. A 20-mm long periodically-poled potassium titanyl phosphate (PPKTP) crystal is pumped by a 405 nm diode laser through type-II spontaneous parametric down-conversion (SPDC) process. The pump light generate orthogonally polarized photons with central wavelength of approximately 810 nm and a FWHM of approximately 0.5 nm. The visibility of polarization entanglement is typically ∼97% (corresponding to a fidelity of 0.98). For the teleportation experiment, we use a pump power of typically 20 mW. The single photon count rate is

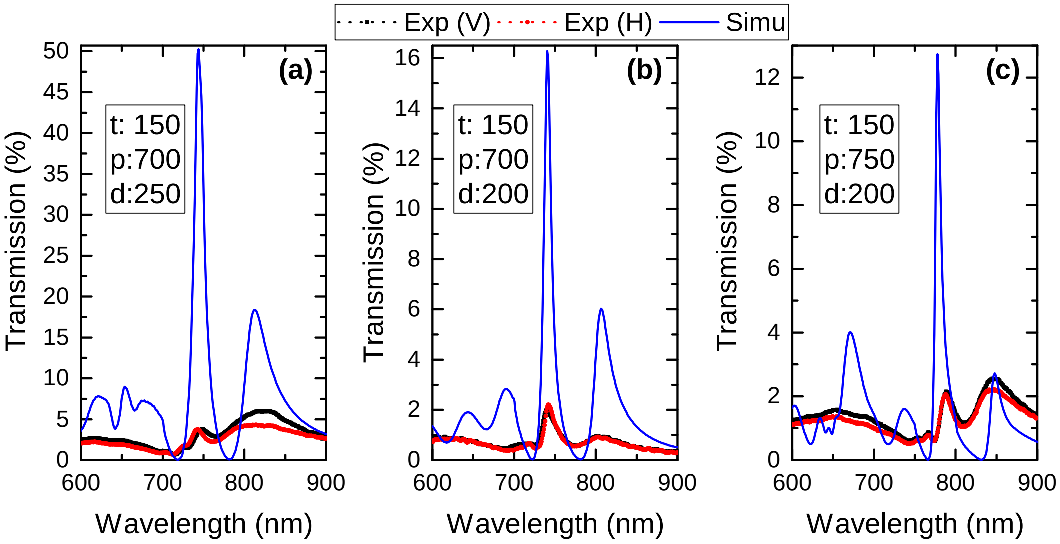

Figure S4. A comparison of the measured and simulated transmission spectra of three samples. (**a**) The transmission spectra for t=150, p=700 and d=250. (**b**) The transmission spectra for t=150, p=700 and d=200. (**c**) The transmission spectra for t=150, p=750 and d=200. Experimental transmission for different polarizations (V and H) are given. The measured peak position of transmission is in agreement with the simulation. The simulated transmittance is general larger than the experiment, which is caused by the imperfect fabrication of our sample. The difference between measurements and simulations are caused by the fabrication errors: the parameters by fabrication departure from the theoretical values and the imperfect junction caused by moving the sample stage.

approximately 0.5 MHz. The coincidence count rate is approximately 0.1 MHz. After one photon passes through the SPP sample, the single and coincidence counts will have a reduction due to the 0.8% transmittance of the sample. After adding all other losses (coupling efficiency $\sim$30%, propagation loss in fibre, EOM, objective, etc, $\sim$30%), the total transmission efficiency of the SPP arm is approximately 0.1%. The BSM arm has an efficiency of approximately 32% (coupling efficiency $\sim$65% and propagation loss $\sim$50%). Therefore, single photon count rate and coincidence count rate with the SPP sample and BSM are approximately 1.5 kHz and 30 Hz (0.1 MHz $\times$ 0.1% $\times$ 32%), respectively.

S4 Certification of quantum properties of SPP

In order to certify that the SPP can preserve the quantum correlation between the generated two photons, we perform the Bell-CHSH inequality tests[7,8]. The violation of this inequality confirms the existence of entanglement between different particles in quantum systems. A hidden variable model requires that:

$$|S| = |E(\theta_1,\theta_2) - E(\theta_1,\theta_2') + E(\theta_1',\theta_2) + E(\theta_1',\theta_2')| \leqslant 2 \tag{S3}$$

where θ_1,θ_2 are angles of the measurement settings corresponding to photon A and B, respectively, and $E(\theta_1,\theta_2)$ is the correlation function with the settings (θ_1,θ_2). The correlation function is defined as

$$E(\theta_1,\theta_2) = \frac{N_{++} - N_{+-} - N_{-+} + N_{--}}{N_{++} + N_{+-} + N_{-+} + N_{--}} \tag{S4}$$

Here, N_{mn} $(m,n = +,-)$ represents the number of detected coincident events with the outcome m for photon A and n for photon B. The measurement settings for the two photons are $(|\theta_1\rangle, |\theta_1'\rangle)$ and $(|\theta_2\rangle, |\theta_2'\rangle)$, respectively. In order to maximally violate the Bell-CHSH inequality, we set the angles of measurement settings to be $\theta_1 = 0°$, $\theta_1' = 45°$, $\theta_2 = 22.5°$ and $\theta_2' = 67.5°$. The number of correlated events for each measurement base are listed in Table **S1**. Our experiment gives a value of $S = 2.551 \pm 0.001$ without the SPP and $S = 2.281 \pm 0.003$ with the SPP, which are well above the classical bound 2. This indicates that the SPP can preserve the quantum correlation of the two photons.

S5 Phase control of Bell-state analyser

Bell-state measurement (BSM) plays a key role in a wide variety of quantum information processes such as entanglement swapping, quantum teleportation and quantum key distribution. Following the Rome scheme[9,10], we utilize both the path

Table S1. The measured coincidence counts N_{mn} for the four base settings: (θ_1, θ_2), (θ_1, θ_2'), (θ_1', θ_2) and (θ_1', θ_2'). A total measurement time of five minutes per setting.

	Without SPP				With SPP			
	N_{++}	N_{+-}	N_{-+}	N_{--}	N_{++}	N_{+-}	N_{-+}	N_{--}
(θ_1, θ_2)	71100	5141100	6729000	275100	10800	842100	1107000	21600
(θ_1, θ_2')	107400	1356300	1763700	1812900	22800	276600	364800	155700
(θ_1', θ_2)	70200	4578600	5300100	413400	16800	664800	798600	54300
(θ_1', θ_2')	42000	6002400	5601000	320400	6300	975300	945300	22500

and polarization degree of freedoms of a single photon to encode the four Bell states to achieve the complete BSM. In our experiment, the four Bell states are defined as follows,

$$|\Psi^{\pm}\rangle_A^{01} = \frac{1}{\sqrt{2}}\left(|V\rangle_A^0|l\rangle_A^1 \pm |H\rangle_A^0|r\rangle_A^1\right)$$
$$|\Phi^{\pm}\rangle_A^{01} = \frac{1}{\sqrt{2}}\left(|H\rangle_A^0|l\rangle_A^1 \pm |V\rangle_A^0|r\rangle_A^1\right) \quad \text{(S5)}$$

After BSM, the state of photon A is projected to one of the four Bell states with equal probability and the state of photon B collapses to the unknown quantum state up to a unitary operation $\{i\sigma_y, \sigma_x, I$ and $\sigma_z\}$, where σ_x , σ_y and σ_z are Pauli matrices. Hence, Alice needs to inform Bob about the outcomes of the BSM in real time via a classical communication channel. Then, Bob carries out the corresponding Pauli matrix operations to recover the original unknown quantum state according to the results of the BSM. The whole setup for the state preparation and BSM are shown in Fig. S5. All the components behind the quarter-wave plate 2 (QWP2) can be regarded as a black box to carry out the complete BSM. To make it clear, we give a detailed description of the BSM process with matrix representation.

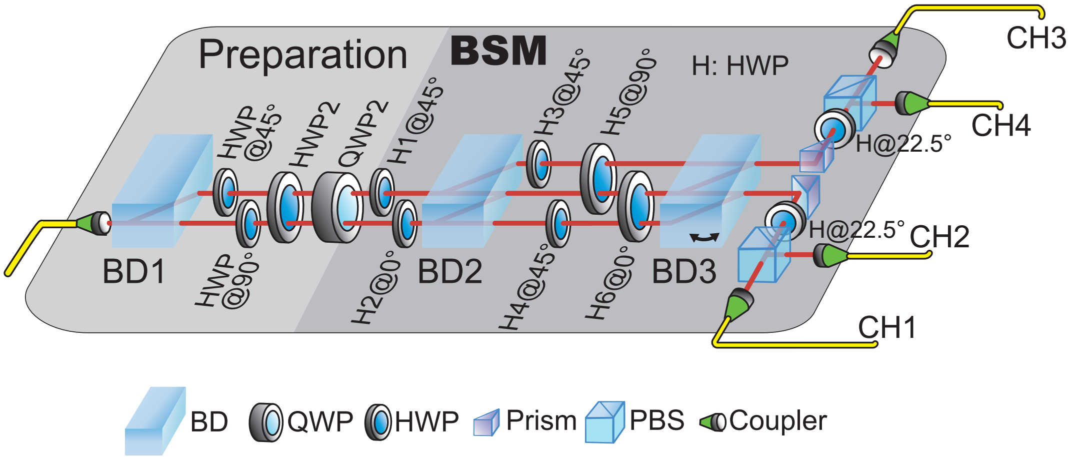

Figure S5. Experimental setup for state preparation and Bell-state measurement (BSM). The path-polarization entangled state $|\Psi^-\rangle_{AB}^{012} = |V\rangle_A^0 \otimes \frac{1}{\sqrt{2}}(|l\rangle_A^0|V\rangle_B^2 - |r\rangle_A^1|H\rangle_B^2)$ is generated after the photon A passes through the BD1, HWP@45° and HWP@90°. There are two wave plates, i.e. HWP2 and QWP2, in both paths to prepare the states to be teleported. The whole setup behind QWP2 is used to realize the complete BSM.

The three path and two polarization modes allow us to construct a 6×6 matrix representation for the transformation of each optical element. Correspondingly, the quantum state can be represented by a vector of six components. The unitary operation matrix of the BSM can be depicted by temporarily disregarding the accumulated phase from each element:

$$\mathbb{U} = \begin{pmatrix} 0 & 0 & 0 & \frac{1}{\sqrt{2}} & -\frac{1}{\sqrt{2}} & 0 \\ 0 & 0 & 0 & -\frac{1}{\sqrt{2}} & -\frac{1}{\sqrt{2}} & 0 \\ 0 & 0 & -\frac{1}{\sqrt{2}} & 0 & 0 & -\frac{1}{\sqrt{2}} \\ 0 & 0 & \frac{1}{\sqrt{2}} & 0 & 0 & -\frac{1}{\sqrt{2}} \\ 0 & 0 & 0 & 0 & 0 & 0 \\ 0 & 0 & 0 & 0 & 0 & 0 \end{pmatrix} \quad \text{(S6)}$$

The four Bell states can be wrote respectively as the following column vectors:

$$|\boldsymbol{\Phi}^{+}\rangle:\begin{pmatrix}0\\0\\1\\0\\0\\1\end{pmatrix},|\boldsymbol{\Phi}^{-}\rangle:\begin{pmatrix}0\\0\\1\\0\\0\\-1\end{pmatrix},|\boldsymbol{\Psi}^{+}\rangle:\begin{pmatrix}0\\0\\0\\1\\1\\0\end{pmatrix},|\boldsymbol{\Psi}^{-}\rangle:\begin{pmatrix}0\\0\\0\\1\\-1\\0\end{pmatrix}$$

When the BSM is finished, the photon A is projected to different Bell states and comes out from individual port. Each port has corresponding vector as follows:

$$\mathbf{CH1}:\begin{pmatrix}0\\0\\1\\0\\0\\0\end{pmatrix},\mathbf{CH2}:\begin{pmatrix}0\\0\\0\\1\\0\\0\end{pmatrix},\mathbf{CH3}:\begin{pmatrix}1\\0\\0\\0\\0\\0\end{pmatrix},\mathbf{CH4}:\begin{pmatrix}0\\1\\0\\0\\0\\0\end{pmatrix}$$

It is not difficult to verify the correspondence of each port to each Bell state by means of matrix operations. In Table S2, we give a detailed list for the mapping of these four ports. From above analysis, we can see that the four Bell states can be fully distinguished using our setup. Therefore, we can in principle perform deterministic teleportation.

Table S2. Exiting port correspondence for the four Bell states.

Ports	$CH1$	$CH2$	$CH3$	$CH4$
Bell states	$\|\Phi^{+}\rangle$	$\|\Phi^{-}\rangle$	$\|\Psi^{-}\rangle$	$\|\Psi^{+}\rangle$

The challenge for our BSM setup is the phase control which includes accumulated relative phase between different paths and phase synchronization of different Bell states $|\Psi^{\pm}\rangle$ and $|\Phi^{\pm}\rangle$. In our experiment, we address the issues by tuning the pitch angle of H3@45° or H4@45°. Before the single photon experiment, classical light with different polarizations is used to calibrate the phase of the BSM interferometer. The interference visibility is optimized to be approximately 96% with a piezoelectric ceramics attached on the BD3 which is driven by an external triangle wave signal. Furthermore, we make a series of phase synchronization test using different incident polarized light (D, A, R, L) with polarizer+HWP+QWP (not shown in Fig. S5) and prepared states (D, A, R, L) with HWP2+QWP2 (see Fig. S5). The results of phase synchronization for seven representative settings (DD, DA, DL, DR, AA, RR, LL, where the first letter denotes the polarization state of incident light and the second letter stands for the prepared state) are given in Figs. S6 and S7. We only need to test the phase synchronization between port CH1 (CH2) and port CH3 (CH4) on account of the instinctive phase identity of CH1 (CH3) and CH2 (CH4) because they go through the same phase difference caused by BD2 and BD3 and exit from the same PBS. The light from the four Bell ports are coupled into SMF and detected with photodetector. By changing the voltage applied on the ceramics, we can scan the phase and obtain the variation of intensity with respect to the phase. From Figs. S6 and S7 we can see that the maximum and minimum almost appear at the same phase location, which indicates that the phase can be synchronized for all the Bell ports using our optimization methods. Note that the intensities of the four Bell ports may have different maximum because of the varying power of incident polarized light and the different coupling efficiencies. Figures S6 and S7 show that the four Bell ports have some symmetric phase relations with respect to each other for different combinations of incident and prepared polarized states. After making the synchronization of four Bell ports, we rotate the azimuth of BD3 to move the phase to the location of maximal contrast (at $\sim\pi$ in Figs. S6 and S7).

S6 Feed-forward unitary transformations

By performing the BSM, four Bell states will be unambiguously discriminated. Then, we need the results of the BSM to be sent from Alice to Bob, who applies the corresponding unitary transformations. The whole feed-forward setup is shown in Fig. S8. The signals from each BSM outcome are used to trigger the electro-optic modulators (EOMs) to implement corresponding Pauli matrix operations. Two EOMs (EOM$_x$: Leysop RTP-X-4-20; EOM$_z$: ConOptics 360-160-4P-LTA) are used to execute the σ_x and σ_z operations, respectively. There are total four unitary operations $\{i\sigma_y, \sigma_x, I, \sigma_z\}$ for the four Bell states. The EOM$_x$ will be triggered to perform the σ_x operation corresponding to the result of $|\Phi^{-}\rangle_A^{01}$ and EOM$_z$ performs the σ_z operation corresponding to the result of $|\Psi^{+}\rangle_A^{01}$. If the outcome of the BSM is $|\Phi^{+}\rangle_A^{01}$, the two EOMs will be triggered simultaneously to perform $i\sigma_y$

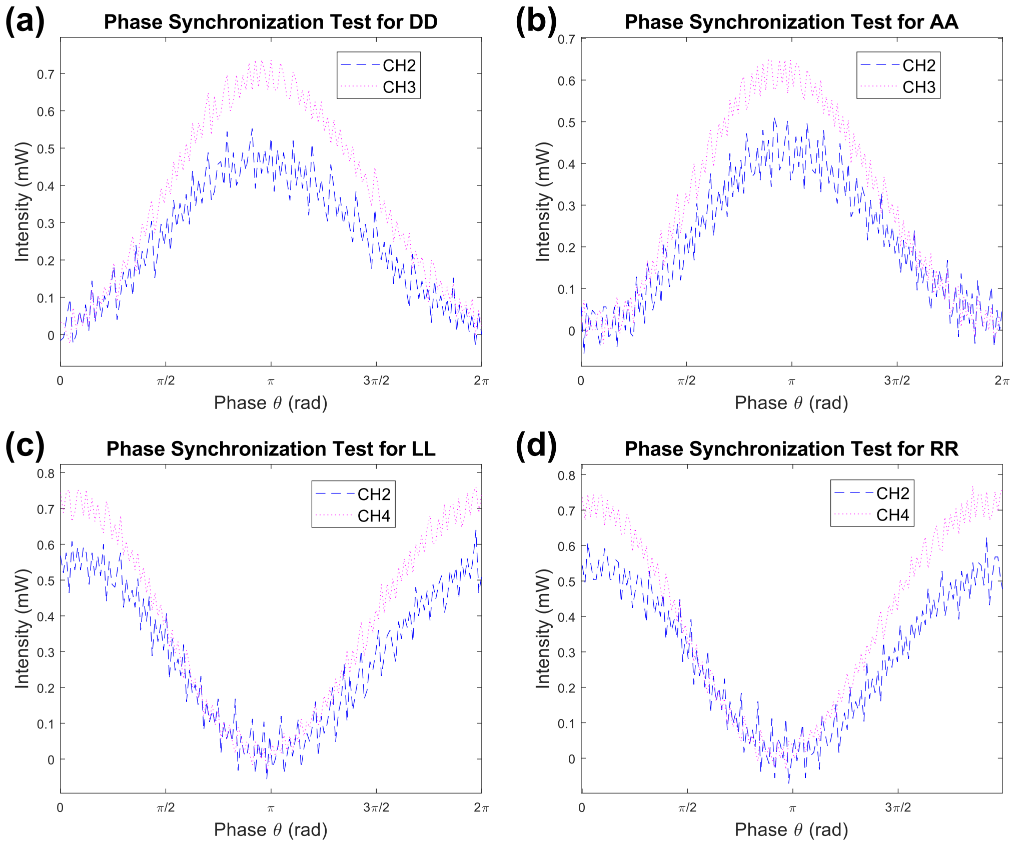

Figure S6. Phase synchronization test for (**a**) DD, (**b**) AA, (**c**) RR and (**d**) LL. The first letter denotes the polarization state of incident light and the second stands for the prepared state. In this case, the incident and prepared states are identical. Through optimization, CH2 is in sync with CH3 for DD and AA and in sync with CH4 for RR and LL. These four tests show the phase synchronization for different incident light polarizations when the polarization of prepared state is the same. D: Diagonal polarization; A: Antidiagonal polarization; R: Right circularly polarization; L: Left circularly polarization.

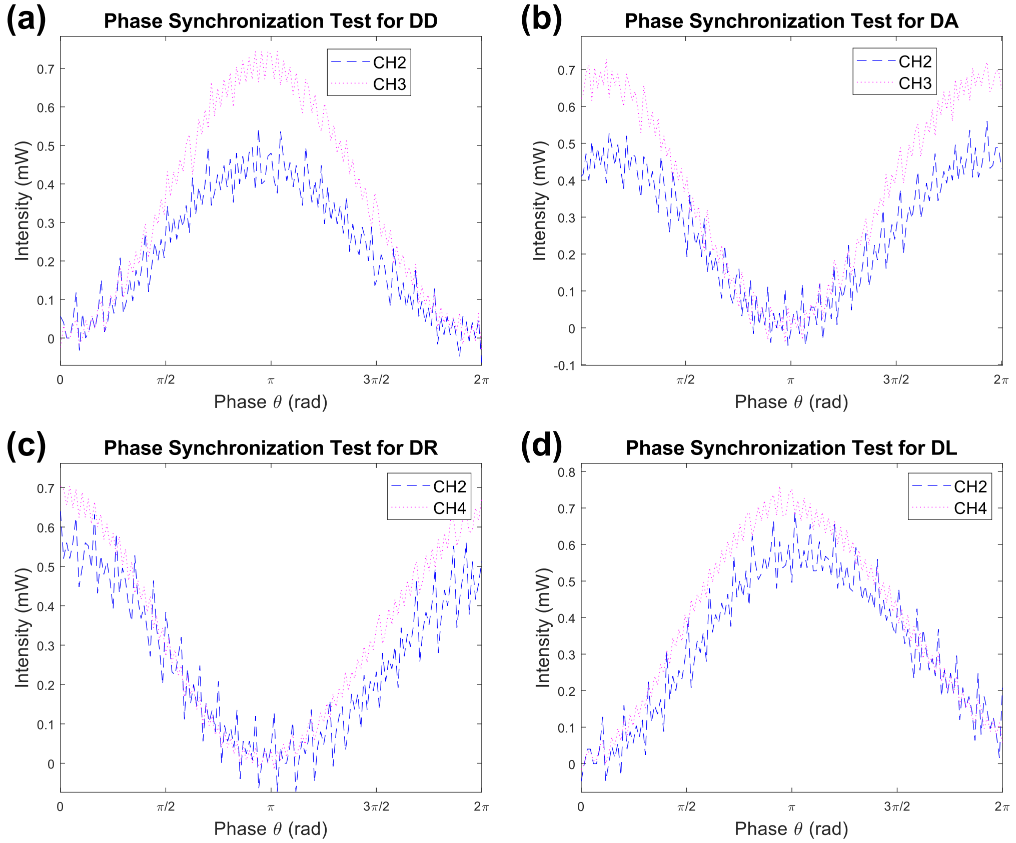

Figure S7. Phase synchronization test for (**a**) DD, (**b**) DA, (**c**) DR and (**d**) DL. The meanings of these letters are the same as in Fig. S6. In this case, the incident state is always D and four prepared states are D, A, R and L, respectively. Note that (**a**) is the same as Fig. S6(a). Through optimization, CH2 is in sync with CH3 for DD and DA and in sync with CH4 for DR and DL. As a complementary part of Fig. S6, these tests can guarantee the perfect phase synchronization for the same incident light with general prepared states.

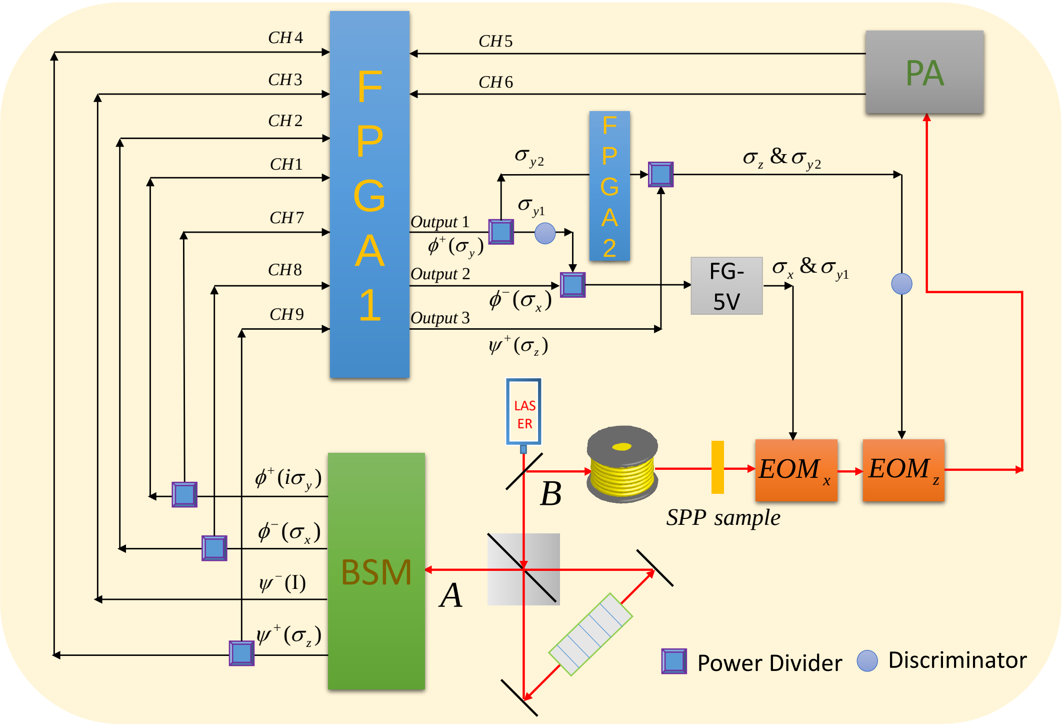

Figure S8. Feed-forward unitary transformation circuit with EOM. The two EOMs perform the Pauli operations acting on photon B to complete the feed-forwrd unitary transformations so as to recover the original quantum states of photon A. The signal of photon A coming out from the BSM is divided into two copies, one is used for coincidence detection with the photon B and another is used for triggering the EOMs. The two FPGAs are exploited to tune the delay of triggering signals acting on the two EOMs. The delay of signal $|\Phi^-\rangle_A^{01}$ from CH8 to Output2 of FPGA1 is tuned to trigger the EOM$_x$. The delay of signal $|\Psi^+\rangle_A^{01}$ from CH9 to Output3 is tuned to trigger the EOM$_z$. The signal $|\Phi^+\rangle_A^{01}$ coming out from Output1 is split into σ_{y1} and σ_{y2} and will trigger EOM$_x$ and EOM$_z$ simultaneously. Note that the FPGA2 is utilized to compensate the delay cost by the function generator (FG). Here, six power dividers are used to divide and combine the signal from different Bell states. The discriminator is used to provide the voltage of external triggering pulse acting on the EOMs. PA: polarization analyser; BSM: Bell-state measurement; FPGA: Field Programmable Gate Array.

operation ($\sigma_z \cdot \sigma_x = i\sigma_y$). Here, field programmable gate arrays (FPGAs) are utilized to tune the delay between photon A and photon B. Because the inner interval of FPGA and electronic delay of trigger signal from FPGA to EOM have a minimum limit (247 ns and 114 ns), we use an extra 222 m (time delay of $\sim$1110 ns) SMF to allow the free adjustment of the delay time. In our experiment, three channels (CH7, CH8, CH9) of FPGA1 are used to set the delay between the detection signal of photon A for each Bell port and the trigger signal acting on photon B. Furthermore, the trigger signal from the Output1 set by CH7 is divided into two paths and used to trigger EOM_x and EOM_z simultaneously. For EOM_x, we need a function generator (FG) to produce a $>$5 V external triggering voltage. Therefore, we add another FPGA2 in the σ_{y2} path to compensate the delay caused by FG. This can put the pulse of the two signals (Output1$\rightarrow \sigma_{y1} \rightarrow \text{EOM}_x$, Output1$\rightarrow \sigma_{y2} \rightarrow \text{EOM}_z$) in the same time window. Through scanning the time of CH7, CH8 and CH9, the delay of trigger signal corresponding to three Bell states ($|\Psi^+\rangle_A^{01}$, $|\Phi^-\rangle_A^{01}$ and $|\Phi^+\rangle_A^{01}$) can be determined, respectively.

In order to obtain a good contrast, we optimize the half-wave voltage of these two EOMs and set the pulse width of trigger signal to be 200 (100) ns for EOM_x (EOM_z). The detailed specifications of these two EOMs are listed in Table S3.

Table S3. Some typical parameters of the two EOMs used in our experiment.

	Half-wave voltage	Fast axis's angle	Pauli matrix	Contrast	Pulse width
EOM_x	1.22 kV	45°	σ_x	77.5	200 ns
EOM_z	70.2 V	0°	σ_z	29.7	100 ns

S7 Reconstructed density matrix and teleportation fidelity

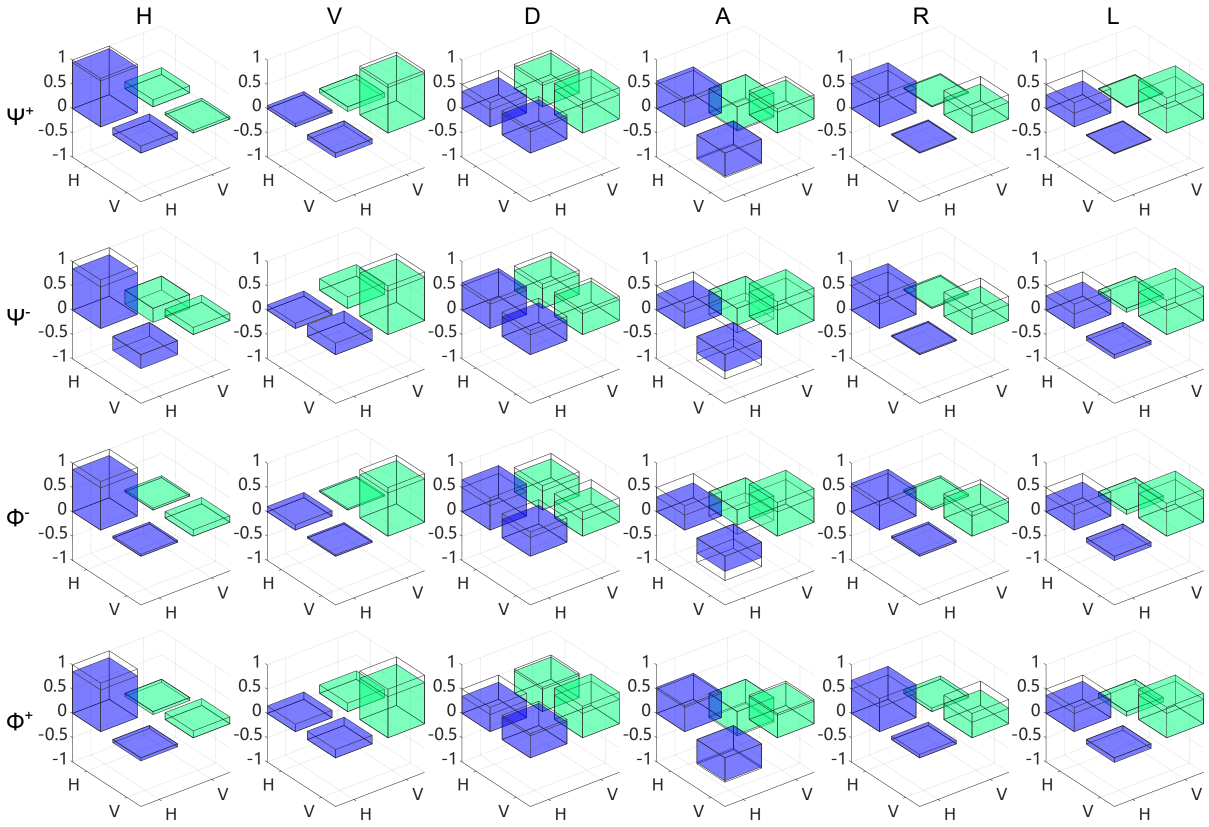

Figure S9. Real parts of the reconstructed density matrices for the six teleported states. The six columns represent the six teleported states and each of the four rows corresponds to one of the possible BSM outcomes. The ideal density matrix $|\phi\rangle_{\text{ideal ideal}}\langle\phi|$ is shown as the wire grid.

We prepare six input states $|H\rangle$, $|V\rangle$, $|D\rangle$, $|A\rangle$, $|R\rangle$ and $|L\rangle$ at the Alice's side. The density matrices for the six teleported quantum states including active feed-forward operations are reconstructed by means of quantum state tomography (QST)[11]. The real and imaginary parts of the reconstructed density matrices for each of the six states corresponding to the four BSM

outcomes are shown in Figs. S9 and S10, respectively. All these figures are for the teleportation with the SPP involved. We can see that the $|H\rangle$ and $|V\rangle$ states have one dominating element. For $|D\rangle$ and $|A\rangle$ states, the four elements have approximate equal weight. The diagonal elements are in opposite sign with the antidiagonal elements for $|A\rangle$ state. For $|R\rangle$ and $|L\rangle$ states, the diagonal elements are real and the antidiagonal elements are imaginary. These features are consistent with the ideal density matrices of these six states.

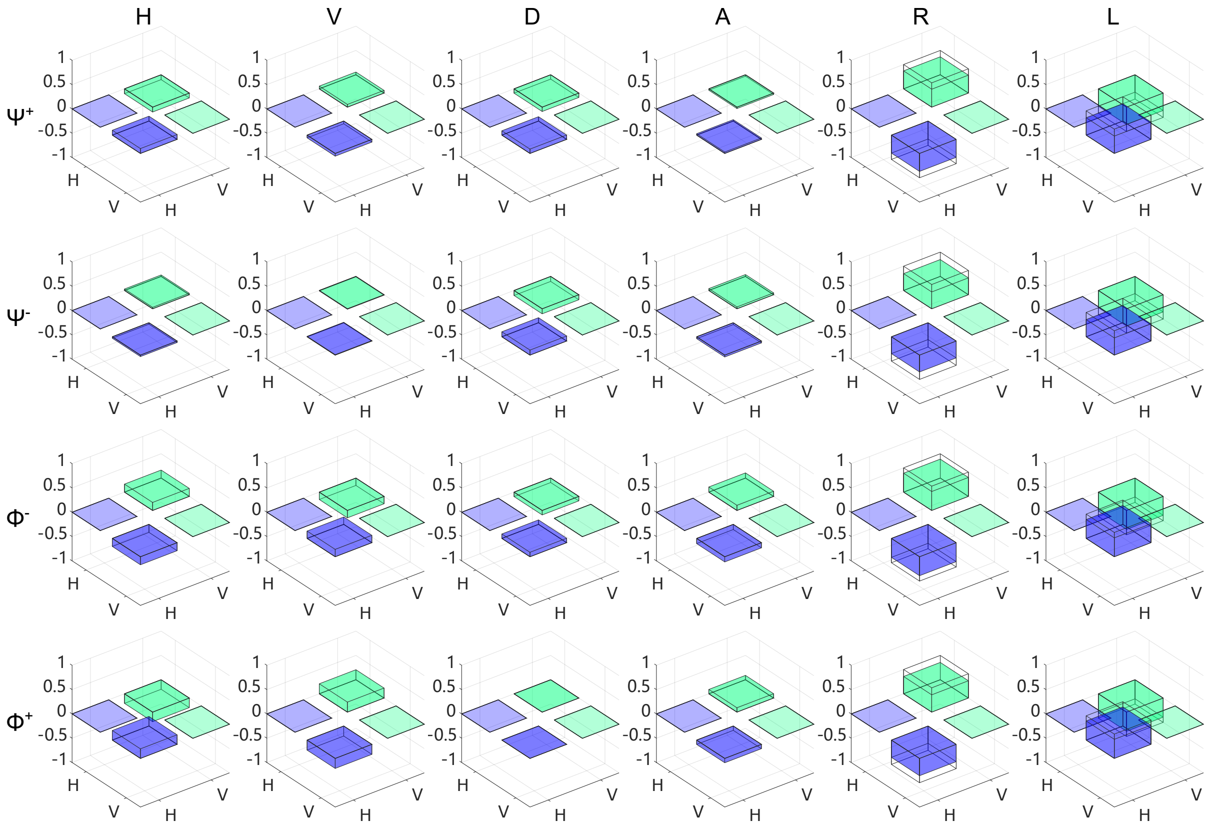

Figure S10. Imaginary parts of the reconstructed density matrices for the six teleported states. Plots are similar to these described in the caption of Fig. S9.

The teleportation fidelities of these states are calculated with the reconstructed density matrices via $F = {}_{\text{ideal}}\langle\phi|\rho|\phi\rangle_{\text{ideal}}$, where $|\phi\rangle_{\text{ideal}}$ is the ideal quantum state. The uncertainties in state fidelities are calculated using a Monte-Carlo method assuming Poissonian counting statistics. In Table S4 and S5, we give the obtained fidelity data for both without (Table S4) and with (Table S5) the SPP. The experimental measured coincidence counts for the teleportation fidelities are shown in Table S6 (without SPP) and S7 (with SPP). The teleported state $|\phi\rangle$ is projected to detectors D5 and D6 for tomography (see Fig. 1(e) in main text). For the states $|H\rangle$, $|D\rangle$ and $|R\rangle$, $|\phi\rangle$ is projected to detector D5 and the orthogonal state $|\phi\rangle^{\perp}$ is projected to D6. For $|V\rangle$, $|A\rangle$ and $|L\rangle$ states, $|\phi\rangle$ is projected to D6 and the orthogonal state $|\phi\rangle^{\perp}$ is projected to D5.

Table S4. State fidelities without SPP (in units of %).

	$\|H\rangle$	$\|V\rangle$	$\|D\rangle$	$\|A\rangle$	$\|R\rangle$	$\|L\rangle$
Ψ^+	95.81±0.27	97.22±0.21	95.17±0.25	95.90±0.25	95.20±0.27	97.98±0.28
Ψ^-	88.22±0.38	91.25±0.36	92.71±0.37	88.37±0.40	93.99±0.26	96.85±0.20
Φ^-	89.47±0.43	91.48±0.39	91.40±0.34	87.50±0.46	92.32±0.36	94.03±0.30
Φ^+	88.02±0.40	86.46±0.42	95.28±0.27	96.83±0.21	90.18±0.29	92.43±0.26

The reduction in state fidelities with SPP compared to that of without (W.O.) SPP can be attributed to different parts of the experiment. The effect of the imperfect optical elements and multiphoton noise can be treated as the white noise and the

Table S5. State fidelities with SPP (in units of %).

	$\|H\rangle$	$\|V\rangle$	$\|D\rangle$	$\|A\rangle$	$\|R\rangle$	$\|L\rangle$
Ψ^+	94.80±0.35	93.62±0.36	93.97±0.33	97.44±0.22	86.95±0.32	85.75±0.23
Ψ^-	85.32±0.50	89.25±0.38	90.55±0.42	84.50±0.47	84.68±0.35	85.34±0.28
Φ^-	86.94±0.55	89.04±0.49	88.64±0.48	81.09±0.73	88.98±0.34	88.88±0.35
Φ^+	85.85±0.44	84.66±0.38	95.67±0.25	97.04±0.22	85.58±0.39	89.31±0.33

Table S6. Experimental measured coincidence counts for state fidelities without SPP (total integration time 60s). Each Bell port is made coincidence with the projected state $|\phi\rangle$ and its orthogonal state $|\phi\rangle^{\perp}$.

$\|\phi\rangle$		$\|H\rangle$	$\|V\rangle$	$\|D\rangle$	$\|A\rangle$	$\|R\rangle$	$\|L\rangle$
Ψ^+	$\|\phi\rangle$	2,746	2,554	2,639	2,552	2,442	2,034
	$\|\phi\rangle^{\perp}$	120	73	134	109	123	42
Ψ^-	$\|\phi\rangle$	2,359	2,525	2,596	2,417	2,206	2,060
	$\|\phi\rangle^{\perp}$	315	242	204	318	141	67
Φ^-	$\|\phi\rangle$	2,124	2,050	2,178	1,988	2,117	1,734
	$\|\phi\rangle^{\perp}$	250	191	205	284	176	110
Φ^+	$\|\phi\rangle$	3,336	3,053	3,794	3,264	2,956	2,967
	$\|\phi\rangle^{\perp}$	454	478	188	107	322	243

Table S7. Experimental measured coincidence counts for state fidelities with SPP (total integration time 900s). Each Bell port is made coincidence with the projected state $|\phi\rangle$ and its orthogonal state $|\phi\rangle^{\perp}$.

$\|\phi\rangle$		$\|H\rangle$	$\|V\rangle$	$\|D\rangle$	$\|A\rangle$	$\|R\rangle$	$\|L\rangle$
Ψ^+	$\|\phi\rangle$	2,205	2,435	2,367	1,980	1,572	1,914
	$\|\phi\rangle^{\perp}$	121	166	152	52	236	318
Ψ^-	$\|\phi\rangle$	1,790	2,515	2,118	2,017	1,890	1,746
	$\|\phi\rangle^{\perp}$	308	303	221	370	342	300
Φ^-	$\|\phi\rangle$	1,677	2,055	1,669	1,509	1,340	1,582
	$\|\phi\rangle^{\perp}$	252	253	214	352	166	198
Φ^+	$\|\phi\rangle$	2,640	3,080	3,579	2,494	2,446	2,172
	$\|\phi\rangle^{\perp}$	435	558	162	76	412	260

generated two-photon states are approximated as the Werner states[12],

$$\rho_1 = F_{source}\,|\Psi^-\rangle_{AB\,AB}\langle\Psi^-| + \frac{1-F_{source}}{4}\mathbf{I} \tag{S7}$$

where $|\Psi^-\rangle_{AB} = \frac{1}{\sqrt{2}}(|HV\rangle - |VH\rangle)$. After the source, photon A enters the BSM and photon B passes through SPP, two EOMs and other optical elements (lens, wave plates, mirror, etc.). We denote the operations as $M = \mathrm{BSM}_A \otimes (\mathrm{OE}\cdot\mathrm{EOMz}\cdot\mathrm{EOMx}\cdot\mathrm{SPP})_B$ (OE stands for other optical elements). Finally, the state becomes $\rho_2 = F_{tot} M \rho_1 M^\dagger + \frac{1-F_{tot}}{4}\mathbf{I}$. The BSM, SPP and two EOMs are responsible for the observed reduction in the measured fidelity and we label their fidelities as F_{BSM}, F_{SPP}, F_{EOMx} and F_{EOMz}. The fidelity reduction caused by remaining optical elements is denoted by F_{OE}. Therefore, the state fidelity including all these components can be expressed as:

$$F_{tot} = F_{source}\cdot F_{BSM}\cdot F_{SPP}\cdot F_{EOMx}\cdot F_{EOMz}\cdot F_{OE} \tag{S8}$$

The non-ideal optical elements (such as PBS, wave plates, mirror and so on) and multiphoton emission reduce the quality of the two-photon entanglement and lead to the 98.34% fidelity of the source. The imperfect settings of HWP and BD limit the visibility of quantum interference of Bell-state analyser and lead to the 97.87% fidelity of BSM. To characterize the influences of feed-forward operations on the fidelity, we directly prepare the six states in the SPP setup and measure the state fidelity for individual EOM when moving in and moving out the SPP. By averaging the fidelities over all input states, we obtain the state fidelities of 94.15% with the SPP and 96.14% without the SPP for EOMx. For EOMz, the average state fidelities are 95.32% with the SPP and 97.64% without the SPP. In addition, we remove the two EOMs and measure the state fidelities both without and with the SPP. This gives the state fidelities of F_{OE} = 98.47% for W.O. SPP and 95.81% for with SPP, respectively. Because all the other optical elements are included during the measurement of two EOMs and SPP, we need to eliminate the fidelity F_{OE} and get the net fidelities for these three components. From the above data, we obtain the average state fidelities of F_{EOMx} = 0.9415/0.9847 = 95.61% (With SPP) and F_{EOMx} = 0.9614/0.9847 = 97.63% (W.O. SPP) for EOMx. The corresponding state fidelities are F_{EOMz} = 0.9532/0.9847 = 96.80% (With SPP) and F_{EOMz} = 0.9764/0.9847 = 99.16% (W.O. SPP) for EOMz. With the SPP involved, the state fidelity is F_{SPP} = 0.9581/0.9847 = 97.30%. Finally, we calculate the average state fidelities for both W.O. SPP and with SPP to be:

$$F_{W.O.\ SPP} = F_{source}\cdot F_{BSM}\cdot F_{EOMx}\cdot F_{EOMz}\cdot F_{OE} = 91.75\% \tag{S9a}$$

$$F_{With\ SPP} = F_{source}\cdot F_{BSM}\cdot F_{SPP}\cdot F_{EOMx}\cdot F_{EOMz}\cdot F_{OE} = 85.35\% \tag{S9b}$$

The above quantitative analysis indicates that the excitation of the SPP mode can lead to the deterioration of the beam pattern, which decreases the modulation contrast of the two EOMs and finally results in the reduction of the state fidelity.

References

1. Ghaemi, H. F., Thio, T., Grupp, D. E., Ebbesen, T. W. & Lezec, H. J. Surface plasmons enhance optical transmission through subwavelength holes. *Phys. Rev. B* **58**, 6779–6782 (1998).
2. Olmon, R. L. *et al.* Optical dielectric function of gold. *Phys. Rev. B* **86**, 235147 (2012).
3. Krishnan, A. *et al.* Evanescently coupled resonance in surface plasmon enhanced transmission. *Opt. Commun.* **200**, 1–7 (2001).
4. Altewischer, E., van Exter, M. P. & Woerdman, J. P. Polarization analysis of propagating surface plasmons in a subwavelength hole array. *J. Opt. Soc. Am. B* **20**, 1927–1931 (2003).
5. Kim, T., Fiorentino, M. & Wong, F. N. C. Phase-stable source of polarization-entangled photons using a polarization sagnac interferometer. *Phys. Rev. A* **73**, 012316 (2006).
6. Fedrizzi, A., Herbst, T., Poppe, A., Jennewein, T. & Zeilinger, A. A wavelength-tunable fiber-coupled source of narrowband entangled photons. *Opt. Express* **15**, 15377–15386 (2007).
7. Bell, J. S. On the einstein podolsky rosen paradox. *Physics Physique Fizika* **1**, 195–200 (1964).
8. Clauser, J. F., Horne, M. A., Shimony, A. & Holt, R. A. Proposed experiment to test local hidden-variable theories. *Phys. Rev. Lett.* **23**, 880–884 (1969).
9. Boschi, D., Branca, S., De Martini, F., Hardy, L. & Popescu, S. Experimental realization of teleporting an unknown pure quantum state via dual classical and Einstein-Podolsky-Rosen channels. *Phys. Rev. Lett.* **80**, 1121–1125 (1998).
10. Jin, X.-M. *et al.* Experimental free-space quantum teleportation. *Nature Photon.* **4**, 376–381 (2010).

11. James, D. F. V., Kwiat, P. G., Munro, W. J. & White, A. G. Measurement of qubits. *Phys. Rev. A* **64**, 052312 (2001).

12. Werner, R. F. Quantum states with einstein-podolsky-rosen correlations admitting a hidden-variable model. *Phys. Rev. A* **40**, 4277–4281 (1989).
